# Pre-therapeutic efficacy of the CDK inhibitor dinaciclib in medulloblastoma cells

**DOI:** 10.1038/s41598-021-84082-3

**Published:** 2021-03-08

**Authors:** Marta Buzzetti, Sonia Morlando, Dimitrios Solomos, Ammara Mehmood, Alexander W. I. Cox, Mattia Chiesa, Yuri D’Alessandra, Michela Garofalo, Caroline H. Topham, Gianpiero Di Leva

**Affiliations:** 1 School of Science, Engineering and Environment, Biomedical Research Centre, Salford, Greater Manchester UK; 2 Transcriptional Networks in Lung Cancer Group, Cancer Research UK Manchester Institute, University of Manchester, Manchester, UK; 3 Bioinformatics and Artificial Intelligence Facility, Centro Cardiologico Monzino IRCCS, Milan, Italy; 4 Immunology and Functional Genomics Unit, Centro Cardiologico Monzino IRCCS, Milan, Italy; 5 Cancer Research UK Lung Cancer Centre of Excellence, at Manchester and University College London, London, UK; 6 School of Pharmacy and Bioengineering, Guy Hilton Research Centre, Keele University, Stoke-on-Trent, UK

**Keywords:** Cancer, Molecular medicine

## Abstract

Medulloblastoma (MB) is the most common aggressive paediatric brain tumour and, despite the recent progress in the treatments of MB patients, there is still an urgent need of complementary or alternative therapeutic options for MB infants. Cyclin Dependent Kinase inhibitors (CDKi) are at the front-line of novel targeted treatments for multiple cancers and the CDK4/6 specific inhibitor palbociclib has been pre-clinically identified as an effective option for MB cells. Herein, we identified the pan-CDKi dinaciclib as a promising alternative to palbociclib for the suppression of MB cells proliferation. We present evidence supporting dinaciclib’s ability to inhibit MB cells in vitro proliferation at considerably lower doses than palbociclib. Sequencing data and pathway analysis suggested that dinaciclib is a potent cell death inducer in MB cells. We found that dinaciclib-triggered apoptosis is triggered by CDK9 inhibition and the resultant reduction in RNA pol II phosphorylation, which leads to the downregulation of the oncogenic marker MYC, and the anti-apoptotic protein MCL-1. Specifically, we demonstrated that MCL-1 is a key apoptotic mediator for MB cells and co-treatment of dinaciclib with BH3 mimetics boosts the therapeutic efficacy of dinaciclib. Together, these findings highlight the potential of multi-CDK inhibition by dinaciclib as an alternative option to CDK4/6 specific inhibition, frequently associated with drug resistance in patients.

## Introduction

Medulloblastoma (MB) is the most common paediatric brain tumour, with an overall survival rate of about 70%, which is even lower for high-risk patients^1^. Gene expression and genomic studies highlighted the molecular complexity of this malignant tumour and allowed MB to broadly be divided into four main subgroups: Wingless (WNT), Sonic Hedgehog (SHH), Group 3 and Group 4^2^. Whilst WNT and SHH subgroups are named after the corresponding aberrant signalling pathways, Group 3 and 4 are less clearly related to a specific molecular alteration and are generally characterised by high levels of N- and C-MYC expression, p53 deletion or mutation and extensive aneuploidy and frequent cytogenetic aberrations^3,4^. Despite these four subgroups have been included in the WHO classification of Central Nervous System tumours^5^, two recent studies have shown that MB tumours manifest a much larger molecularly heterogeneity and they can be reliably sub-classified into multiple subtypes by using their DNA methylation patterns or gene expression profiles^4^. Interestingly, both reports agreed that Group 3 MB tumours with MYC amplification or overexpression have a dismal outcome and the poorest prognosis, highlighting that it is of a paramount importance to tailor MB treatments to its biology. However, current MB standard of care includes a generalised combination of surgical resection, chemotherapy and cranio-spinal radiotherapy^6^. These approaches do not account for the intra- and inter-heterogeneity of the MB tumours, leading to significant treatment-associated sequelae and suboptimal survival rates. Thus, there is a pressing need in the clinic for more molecularly tailored therapies for MB, which would allow drug dosage reduction and improvement of patient’s quality of life.

A multi-faceted dysregulation of cell cycle progression mechanisms, such as the unrestrained activation of cyclin-dependent kinases (CDKs), is a common feature of almost all human tumours, including MB^7,8^. CDKs are a class of serine/threonine kinases best known as critical players in the control of the cell cycle progression, a role mainly performed by CDK1, CDK2, CDK4 and CDK6^9^. Nonetheless, other CDKs have non-cell cycle related functions and are involved in transcription (i.e. CDK7, CDK9, CDK12 and CDK13) and several other cellular processes, such as the atypical CDK5 with its role in neuronal development^10,11^. Given the widespread implications of CDKs in functions frequently found abnormal in cancer, there has been a growing effort in developing small inhibitors targeting CDKs for cancer treatment.

In the past few years, drug design approaches mainly focused on the development of selective CDK inhibitors (CDKis), specifically targeting CDK4/CDK6/cyclin D complexes to control highly proliferating cells by blocking only G1/S phase, and in an attempt to limit the side-effects of broad CDK inhibition^12^. Palbociclib is the first CDK4/6 inhibitor approved for advanced breast cancer treatment and effective against many other solid tumours^13^. After showing promising inhibitory effects against Group 3 and SHH MB tumours, palbociclib is the only CDKi selected for MB treatment in clinical trials^14^. Nonetheless, this specific CDK4/6 inhibitor has been associated exclusively with a cytostatic effect and multiple mechanisms of resistance in patients have been uncovered^15^.

Furthermore, considering that CDKs are dispensable and can compensate for each other's function^16^, we speculated that truly specific CDK inhibition, as with CDK4/6 inhibitors, might not be the best option considering the high adaptability of cancer cells and the quick onset of compensatory mechanisms after treatments. More potent CDKis than specific CDK4/6 inhibitors might already be available, and a successful therapeutic application might be more dependent on the optimization of administration schedule and sequence with the current standard of care.

In this study, we investigated the efficacy of a set of currently available CDKis on in vitro models of MB and identified the broad CDKi dinaciclib as a more valuable cytotoxic option than CDK4/6 cytostatic inhibition for MB treatment. Furthermore, to our knowledge, this represents one of the first studies unfolding the mechanism of action for dinaciclib in medulloblastoma and evaluating its applicability for combination therapies in this context.

## Results

### Dinaciclib outperforms CDK4/6 inhibitors in suppressing in vitro proliferation of medulloblastoma cells

**Figure 1:**
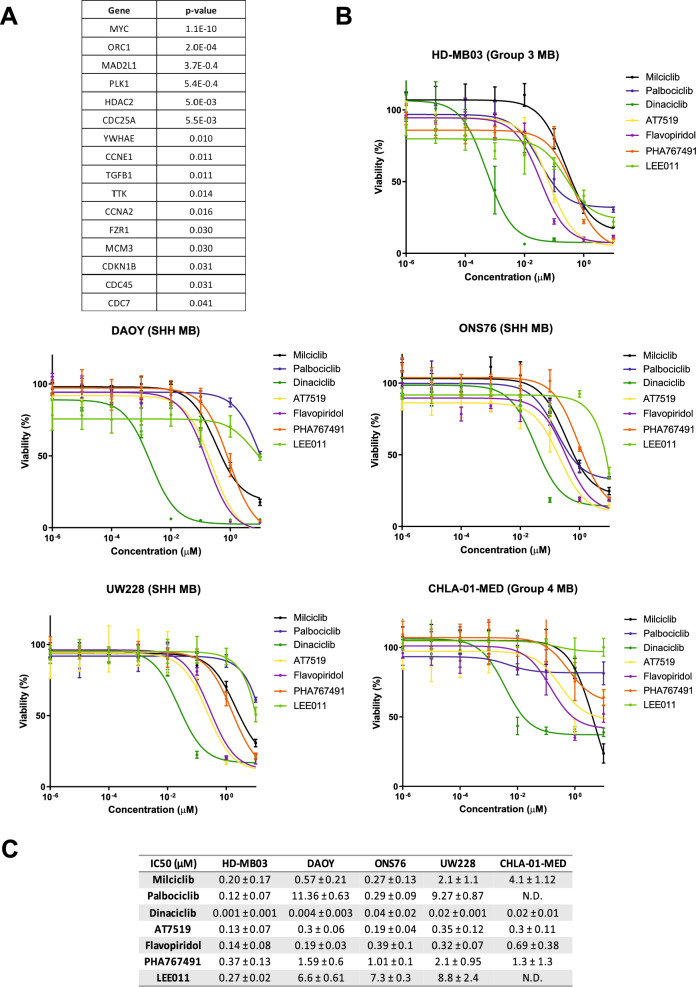
Screening of CDKis with different selectivity in medulloblastoma in vitro models. (**A**) Transcriptional data associated with the cell cycle gene set from 612 patients across the four MB subgroups (Cavalli et al. dataset). Results were corrected for multiple gene testing by FDR (*P* value < 0.05). (**B**) Proliferation assay of indicated HD-MB03 (Group 3), DAOY (SHH), ONS76 (SHH), UW228 (SHH) and CHLA-01-MED (Group 4) cell lines treated with scalar concentrations (0–10 µM) of indicated CDKis for 72 h. Plotted graphs show mean ± SD (n = 3). (**C**) IC50 values of tested CDKis on medulloblastoma cell lines. IC50 values represent mean ± SD (n = 3).

To identify druggable vulnerabilities in CDK proteins of MB cells, we first screened all significantly altered cell cycle-regulated genes in MB tumours that are positively associated to the survival of MB patients. To this aim, by using the R2 Kaplan Meier Scanner Pro tools^17^, we analysed the expression and survival data of 612 MB patient samples from the Cavalli et al. dataset^18^. Out of the 124 cell cycle-related genes, we found that only 16 genes were significantly modulated (FDR < 0.05) across the different MB subgroups and associated with patient’s survival (Fig. 1A). The overexpression of the transcription factor MYC and its transcriptional program (e.g. CDKN1 and CDC25A) were identified as the most crucial negative prognostic markers across the MB subgroups. Despite no CDK gene being directly identified from our analysis, the expression of direct modulators of CDK2 (i.e. CDKN1 and CCNE1) and CDK1 (i.e. CCNA2, CDC25A and PLK1) activities were also associated to the survival of MB patients. Thus, we decided to compare the anti-proliferative activity of a panel of clinically relevant pan-CDKis, with different CDK specificity and selectivity, including CDK1 and CDK2, to the selective CDK4/6 inhibitors, palbociclib and LEE011 (Supplementary Fig. S1). By using MTT proliferation assays on a set of MB cell lines representative of different MB molecular subgroups, we found that broad CDK inhibitors showed a higher anti-proliferative activity than palbociclib and LEE011 (Fig. 1B). Specifically, dinaciclib, a potent CDK1, CDK2, CDK5 and CDK9 inhibitor, resulted to be the most effective compound across the five different MB cell lines, with an IC50 ranging between 1 and 40 nM (Fig. 1C). CDK inhibition by dinaciclib decreased cell growth in a dose-dependent manner, and the lowest IC50 was observed by the MYC-overexpressing Group 3 HD-MB03 cells (Fig. 1C). Palbociclib has been reported to be selectively active against Group 3 and SHH MB cells^19^. However, although palbociclib and LEE011 were both effective in suppressing the proliferation of HD-MB03 cells at concentrations ranging between 100 and 300 nM, only limited anti-proliferative effects were observed in two out of the three SHH MB cell lines (UW228 and DAOY) (Fig. 1B,C). Moreover, palbociclib only impaired HD-MB03 cell proliferation no more than 70% compared to the controls even at the highest dose of the drug (Fig. 1B,C). Altogether, those data show that pan-CDK inhibitors are more effective in inhibiting the proliferation of all MB cells than specific CDK4/6 inhibitors and that dinaciclib is the drug with the highest anti-proliferative activity against MYC-overexpressing HD-MB03 cells.

### Differential anti-proliferative effects of dinaciclib and palbociclib on HD-MB03 cells

**Figure 2:**
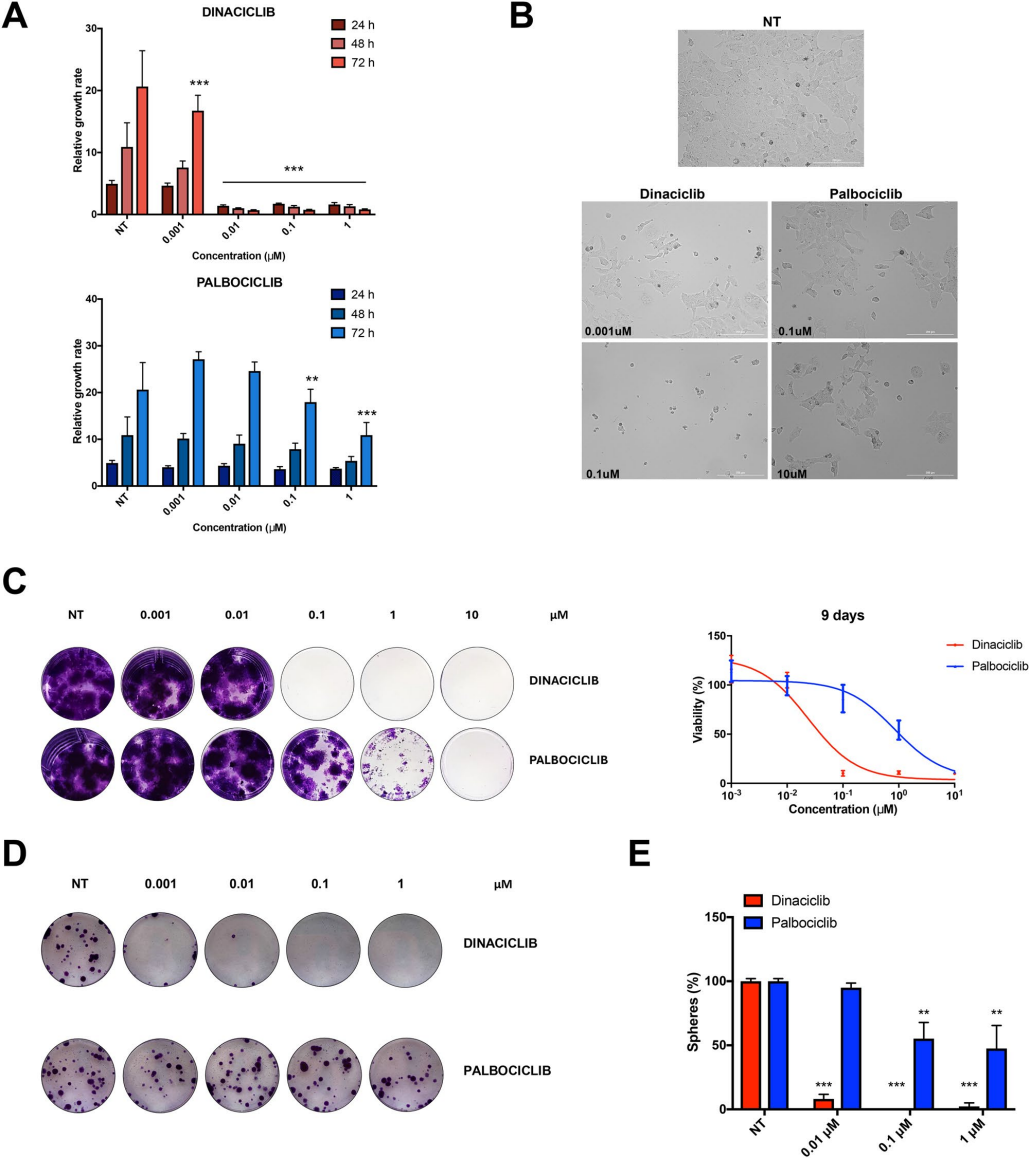
Dinaciclib shows stronger inhibitory responses than palbociclib in medulloblastoma. (**A**) Growth rates over 72 h of HD-MB03 cells treated with dinaciclib (top) or palbociclib (bottom) at the indicated concentrations. (**B**) Representative phase-contrast images of HD-MB03 cells treated with IC50 and 100xIC50 doses of dinaciclib and palbociclib for 72 h. Images were captured at 100 × total magnification. (**C**) Representative crystal violet staining images of long-term proliferation assay. HD-MB03 cells were treated with the indicated drugs for 9 days and then fixed and stained. On the right, crystal violet quantifications relative to the presented images. (**D**) Representative crystal violet staining images showing resistant HD-MB03 colonies arising after dinaciclib and palbociclib wash-out over 12 days recovery. Pre-treatments with each drug were carried out over 24 h before wash-out. (**E**) Percentages of cell viability inhibition of HD-MB03 medullospheres after 72 h treatment with different doses of dinaciclib or palbociclib, calculated as relative to untreated control. Statistical comparisons were performed using an unpaired, two-tailed Student t-test where **P < 0.01; ***P < 0.001. Plotted graphs show mean ± SD (n = 3).

Next, we decided to focus on the pharmacological responses to dinaciclib and palbociclib of HD-MB03 cells, the most sensitive identified cell line to both drugs. First, we assessed the time-dependent inhibitory effects of dinaciclib and palbociclib over the course of 72 h treatment on HD-MB03 cells. The suppressive effects of dinaciclib on cell doubling were already recorded after 24 h of drug treatment at concentrations higher than 10 nM (Fig. 2A). By comparison, palbociclib was 100 times less potent than dinaciclib, causing a significant inhibitory effect on cell proliferation only after 72 h (Fig. 2A). In addition, microscopy analysis of HD-MB03 cells treated with dinaciclib and palbociclib showed that whilst HD-MB03 cells were still alive after treatments with palbociclib, they showed evident accumulation of cell fragments and apoptotic bodies when treated with dinaciclib (Fig. 2B).

The ability of dinaciclib to kill HD-MB03 cells was further assessed by performing repetitive drug treatments of HD-MB03 cells with scalar concentrations of the drugs over a period of 9 days. We found that multiple treatments of HD-MB03 cells with dinaciclib caused complete eradication of MB cells with a 100-fold lower dosage of the drug compared to palbociclib (Fig. 2C). Of note, we were able to kill all HD-MB03 cells with palbociclib only after three consecutive 10 μM drug treatments (Fig. 2C). In parallel, HD-MB03 cells were treated for 24 h with increasing doses of both CDKis, after washing out the drug the survivor cells were then left growing for 2 weeks to form colonies. Again, dinaciclib caused a marked reduction in the number of colonies even at sub-nanomolar concentrations of the drug (0.0001 μM), whilst palbociclib limitedly affected the number of surviving colonies (Fig. 2D).

Because of this ability of dinaciclib to halt the colony formation of HD-MB03 cells, we also assessed the killing effects of both CDKis on the MB cancer stem cell population, a tumour component that is crucial for MB initiation and progression^20^. To this aim, we exposed HD-MB03 cells to either dinaciclib or palbociclib and after 24 h of treatment viable cells were re-seeded into low-attachment culture vessels to promote medullosphere formation. We found that pre-treatments with dinaciclib drastically decreased medullosphere formation whilst exposures to palbociclib resulted only in a 47% reduction in sphere count, even at the highest concentration of palbociclib (Fig. 2E). Overall, dinaciclib shows the ability to suppress the proliferation of HD-MB03 cells at nanomolar concentrations and in a time-dependent manner and profoundly affects the stem-cell like features of this medulloblastoma cell line.

### Dinaciclib induces apoptosis in medulloblastoma cells

**Figure 3:**
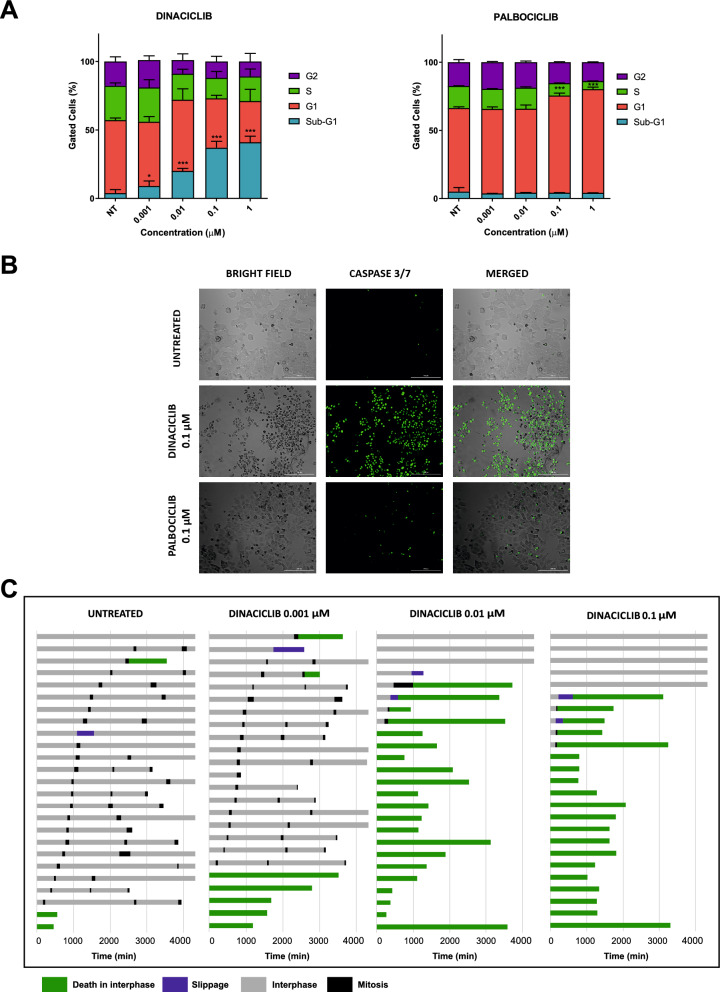
Dinaciclib induces strong cytotoxic effects through apoptosis in medulloblastoma. (**A**) Histogram represents the percentage of HD-MB03 cells arrested in different phases of cell cycle after 24 h treatment with dinaciclib or palbociclib with the indicated doses. (**B**) HD-MB03 cells were treated with indicated drugs for 24 h. CellEvent Caspase 3/7 Green detection reagent was added to live cells and fluorescent signal of apoptotic cells (green) was detected at 100 × total magnification. Representative images are shown. (**C**) Representative cell fate profiles of HD-MB03 cells treated with scalar concentrations of dinaciclib for 72 h. Each line depicts the fate of one single cell followed over time using time-lapse microscopy imaging at 100 × magnification. 0 min is when imaging started. Statistical comparisons were performed using an unpaired, two-tailed Student t-test where *P < 0.05; ***P < 0.001. Plotted graphs show mean ± SD (n = 3).

To better define the underlying causes of the decrease in cell viability of HD-MB03 cells upon dinaciclib treatments and the differential response to palbociclib, we further assessed the cell cycle distribution and apoptotic induction upon 24 h treatment with both CDKis. Treatments with dinaciclib resulted in a significant dose-dependent increase in the sub-G1 cell population as compared to the untreated control (Fig. 3A); treatments with 1 μM dinaciclib induced a 40% accumulation in sub-G1, highlighting the activation of a strong apoptotic response and cytotoxic activity. Conversely, cells treated with palbociclib showed no alteration of the sub-G1 and a main 15% arrest in G0/G1, even at concentrations 10 times the IC50, indicating a cytostatic response (Fig. 3A). To corroborate these results, we performed a caspase 3/7 detection assay that confirmed a selective and robust caspase 3/7 activation only with dinaciclib treatment of HD-MB03 cells (Fig. 3B). This effect was further validated by measuring the cleavage of poly(ADP ribose) polymerase 1 (PARP1) following drug treatment, a common marker of apoptotic induction via caspase-3 activation^21^. We found that only dinaciclib induced PARP1 cleavage in HD-MB03 cells after 24 h treatments, starting from a 0.1 μM dose (Supplementary Fig. S2).

Considering that dinaciclib is able to suppress the activity of multiple CDKs, including the cell cycle related CDK2 and CDK1 that respectively control the G1/S transition and the progression through mitosis^22,23^, we next used a single-cell-based time-lapse microscopy approach to investigate when dinaciclib blocked the cell cycle and induced cell death. To this aim, asynchronous HD-MB03 cells were treated with dinaciclib for 72 h and then 24 randomly selected cells were tracked for their cell cycle progression and death. First, we found that untreated HD-MB03 cells underwent three mitoses during the 72 h period of the experiment with 12% of cells dying in interphase (3 out of 24 cells) and 4% never entering mitosis (Fig. 3C). In the presence of dinaciclib at the lowest tested concentration (1 nM), we observed a 17% increase of cell death in interphase that further increased with higher concentrations of the drug. Specifically, 60% of the cells treated with dinaciclib at 100 nM died in interphase without entering mitosis (14 out of 24 cells), whilst 20% completed mitosis but then died in the following interphase (5 out of 24 cells); the remaining 20% cells never entered mitosis (Fig. 3C). Taken together, our observations show that dinaciclib induces a cytotoxic effect in HD-MB03 cells and it relates to a strong apoptotic response that is activated mainly during interphase.

### Dinaciclib halts the growth of HD-MB03 spheroids by stimulating apoptosis

**Figure 4:**
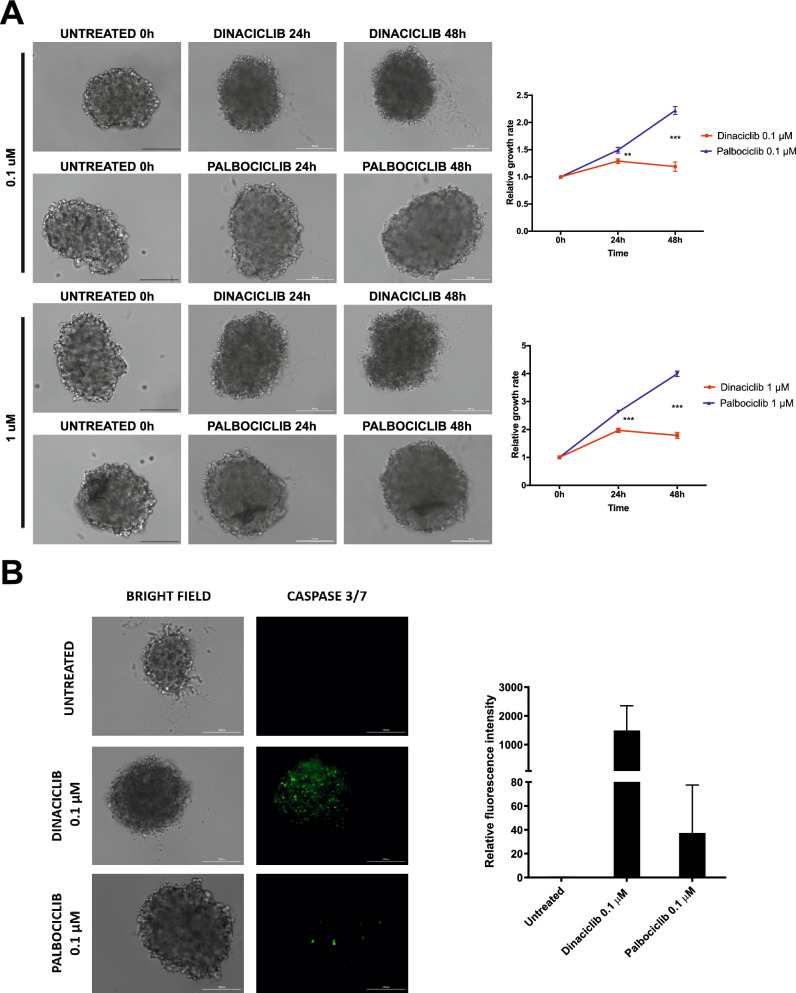
Dinaciclib elicits strong inhibitory effects in 3D medullospheroids and comparable to 2D settings. (**A**) Representative phase-contrast images of HD-MB03 spheroids treated with indicated concentrations of dinaciclib or palbociclib. Each spheroid was imaged before treatment at time 0, at 24 h and 48 h after treatment at 100 × magnification. Scale bar = 200 µm. On the right, relative growth rates values of presented images relative to respective time 0. (**B**) HD-MB03 spheroids were treated with dinaciclib or palbociclib for 24 h. CellEvent Caspase 3/7 Green detection reagent was added to live cells and fluorescent signal of apoptotic cells (green) was detected at 100 × total magnification. Representative images are shown. On the right, fluorescent signal quantifications are relative to the untreated control. Statistical comparisons were performed using an unpaired, two-tailed Student t-test where **P < 0.01; ***P < 0.001. Plotted graphs show mean ± SD (n = 4 technical replicates).

To further evaluate the effect of dinaciclib on HD-MB03 cells in a model that is a reliable predictor of therapeutic efficacy in vivo^24,25^, we employed three dimensional (3D) tumour spheroid cultures of HD-MB03 cells and evaluated the anti-proliferative activity of dinaciclib and palbociclib. Here, HD-MB03 cells were plated and spun in a round bottom 96-well plate to allow the formation of cellular aggregates and mimic the structural properties and physiological interactions of MB cells in tumours. Five days after plating, spheroids were treated with both drugs for 48 h at two different concentrations. The size of the aggregates was measured over time and was reported relative to time 0 h. We found that dinaciclib halted the growth of the spheroids and that they shrunk slightly after 48 h of treatments at both concentrations (Fig. 4A). Upon closer examination, shape of the HD-MB03 spheroids was maintained but cells appeared to die both at the periphery and the core of the spheroids. Conversely, palbociclib was unable to stop the growth of the spheroids and morphological analysis did not reveal any structural changes or accumulation of dead cells (Fig. 4A); dark necrotic areas were only observed within the centre of the spheroids at 48 h with 1 µM palbociclib treatment.

Dinaciclib treatments in 2D cultures were associated with anti-proliferative effects due to a robust apoptotic response. To examine if dinaciclib exerted the same effect in 3D culture of HD-MB03 cells, we measured the caspase 3/7 activation after 24 h of dinaciclib and palbociclib treatments. We observed that spheroids exposed to dinaciclib showed a large number of caspase-positive cells in all treated spheroids, revealing the clear cytotoxic potential of dinaciclib on medulloblastoma cells (Fig. 4B). Moreover, apoptotic cells were diffuse on the full mass of the spheroids, possibly due to the diffusion of the drugs and high levels of cellular stress inside the 3D structure (Fig. 4B). On the contrary, palbociclib did not stimulate the activation of the caspase 3/7 system, showing again a limited apoptotic effect against medulloblasstoma cells.

### Transcriptional CDK9 mediates the anti-proliferative and apoptotic effects of dinaciclib

**Figure 5:**
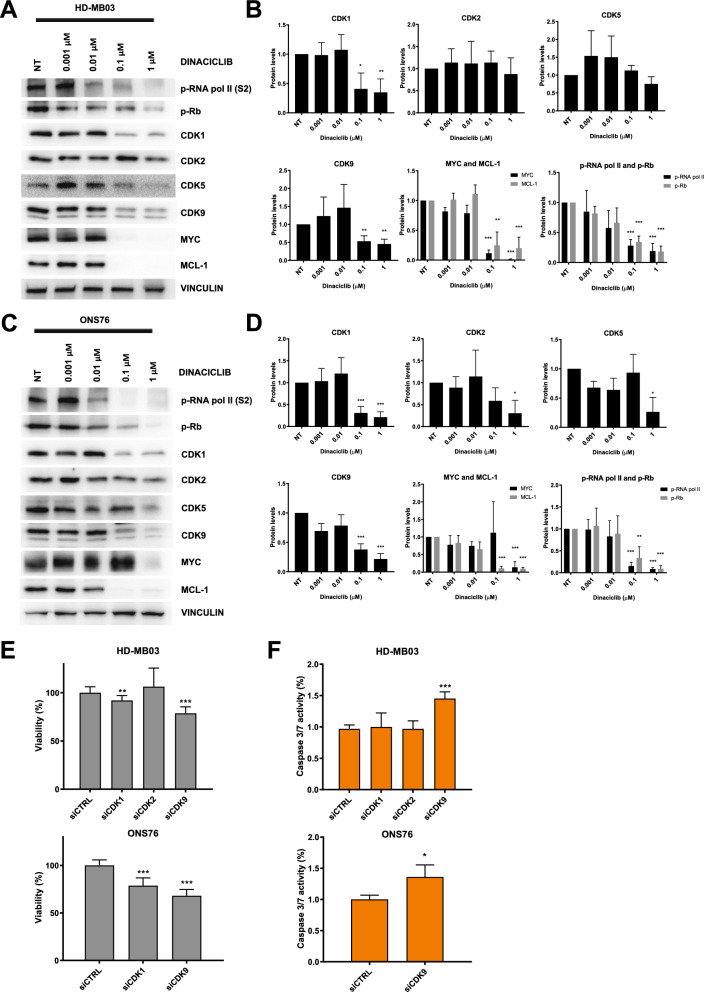
Mediators of cytotoxic effects by dinaciclib in medulloblastoma. (**A**) HD-MB03 cells were treated with indicated doses of dinaciclib for 24 h. Cell extracts were analysed by Western blotting. (**B**) Densitometric analysis of blots presented in (**A**) the other original blots used for the analyses can be found in Supplementary Fig. S3A. (**C**) ONS76 cells were treated with indicated doses of dinaciclib for 24 h. Cell extracts were analysed by Western blotting. (**D**) Densitometric analysis of blots presented in C) the other original blots used for the analyses can be found in Supplementary Fig. S3B. (**E**) Proliferation assay of HD-MB03 and ONS76 cells transfected with siCDK1, siCDK2 or siCDK9 relative to untransfected control (siCTRL). (**F)** Caspase 3/7 activity in HD-MB03 and ONS76 cells treated as above. Values are relative to untransfected control. Statistical comparisons were performed using an unpaired, two-tailed Student t-test where *P < 0.05; **P < 0.01; ***P < 0.001. Plotted graphs show mean ± SD (n = 3).

Next, we aimed to understand the molecular mediators for the cell growth inhibition and apoptotic effects induced by dinaciclib. To this purpose, we first treated two medulloblastoma cell lines with different concentrations of dinaciclib for 24 h and then assessed changes to multiple downstream players of the CDK activities targeted by the drug. Considering the efficacy of dinaciclib in inhibiting both cell cycle and transcriptional CDKs, we mainly detected a decrease in: (1) the phosphorylation levels of pRB, which is a downstream target of CDK2, confirming the suppression of its activity; (2) phosphorylation of the serine 2 of the RNA polymerase II (RNA pol II), a direct target of the transcriptional activity of CDK9. Interestingly, the reduction of CDK9 activity was further confirmed by analysing the reductions of known RNA pol II downstream targets, such as MYC and MCL-1 (Fig. 5A–D and Supplementary Fig. S3A-B), which are promptly influenced by RNA pol II alterations due to their short half-life^26–28^. We also found that dinaciclib induced a marked reduction in the protein levels of the transcriptional CDK9 and cell cycle related CDK1 (Fig. 5A–D and Supplementary Fig. S3A-B).

To further study the direct contribution of CDK1, 2, and 9 targeting to dinaciclib cytotoxic activity in MB cells, we silenced the three CDKs by using a pool of 4 different siRNAs. Upon 72 h silencing of each individual CDK, we evaluated that CDK1 and CDK9 knockdown negatively affected the proliferation of HD-MB03 cells (Fig. 5E and Supplementary Fig. S3C). Conversely, the apoptotic effect, as assessed by caspase 3/7 activity measurements, was exclusively related to CDK9 knockdown (Fig. 5F and Supplementary Fig. S3C). We also confirmed the anti-proliferative effect of CDK1 and 9 and the pro-apoptotic activity of CDK9 in ONS76 cells after knockdown of CDK1 and CDK9 (Fig. 5E,F and Supplementary Fig. S3C).

Overall, these results offer a mechanistic implication of the transcriptional CDK9 and to a lesser extent of the cell cycle related CDK1 in the phenotypic effects observed after in vitro treatments with dinaciclib on medulloblastoma cells.

### BH3-mimetics enhance dinaciclib cell death effects in medulloblastoma

**Figure 6:**
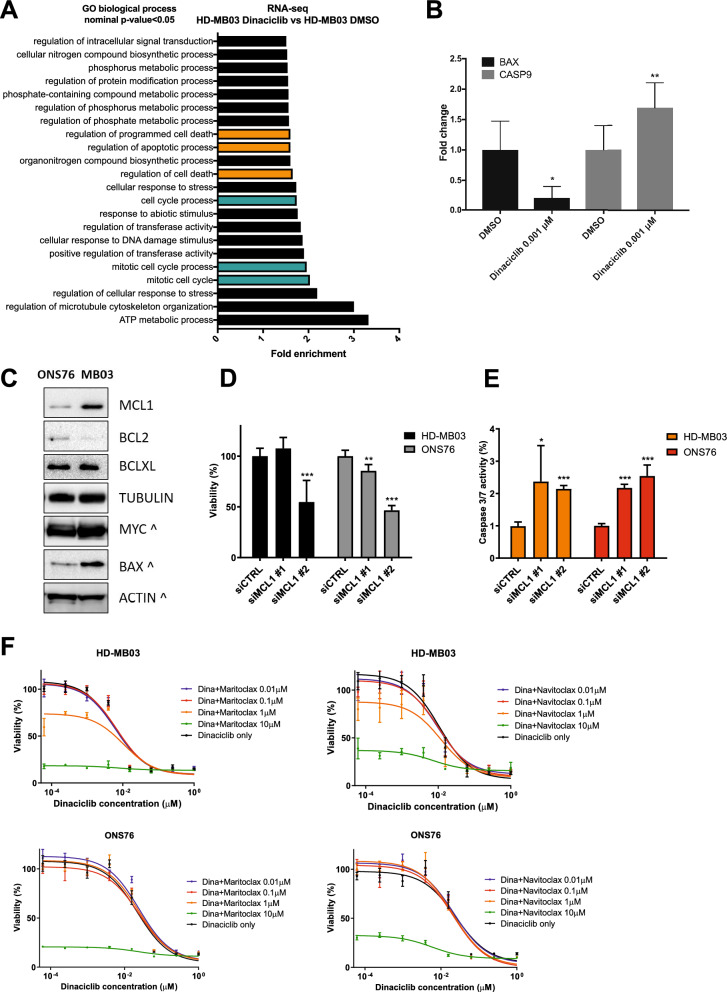
BCL2 family role in apoptosis triggered by dinaciclib in medulloblastoma. (**A**) Gene ontology analysis of RNAseq data performed in HD-MB03 untreated cells versus HD-MB03 cells treated with 1 nM dinaciclib for 24 h using the GO enrichment analysis tool (http://geneontology.org/, release date June 2020). Analysis was performed using significant altered genes with FC > 1.2 and *P* value < 0.05. Biological function terms with fold enrichment score ± 1.5 and FDR < 0.05 are presented. Most relevant functions for the study are highlighted with colours. (**B**) RNAseq validation by single qRT-PCR of BAX and CASP9 in HD-MB03 cells treated with 1 nM dinaciclib or DMSO for 24 h. The graph represents the mean fold change ± SD. (**C**) Representative western blots showing expression patterns of apoptotic proteins in ONS76 and HD-MB03 cells. Tubulin and actin were used as loading controls. ^ symbol indicates that the same proteins were run on a different blot but using the same lysate quantity. (**D**,**E**) Proliferation and caspase 3/7 assays of HD-MB03 and ONS76 cells transfected with siCDK1, siCDK2 or siCDK9 relative to untransfected control. (**F**) Proliferation assays of HD-MB03 and ONS76 cells treated for 72 h with combinations of dinaciclib and maritoclax or navitoclax as indicated. Statistical comparisons were performed using an unpaired, two-tailed Student t-test where *P < 0.05; **P < 0.01; ***P < 0.001. Plotted graphs show mean ± SD (n = 3).

Finally, we performed an RNA sequencing (RNA-seq) analysis comparing the transcriptome of untreated versus dinaciclib-treated HD-MB03 cells. Due to the high potency of the drug and with the purpose of identifying only the most critical response genes, we decided to treat cells with a very low concentration of dinaciclib (1 nM) for a short amount of time (24 h). We identified a set of 197 protein coding genes (92 upregulated, 105 downregulated; *P* value < 0.05, fold change > 1.2) (Supplementary Fig. S4A and Supplementary Table S1) that GO-biological processes analysis revealed to be mainly involved with metabolism, cellular response to stress, cell cycle and programmed cell death (Fig. 6A). We decided to focus exclusively on the validation of genes involved in cell death and found that CASP9 and BAX, effector and mediator of the mitochondrial apoptotic pathway^29,30^, were among the most modulated genes after dinaciclib treatments (Fig. 6B). Since other members of the BCL-2 family (e.g. BAD and BAX, *P* value < 0.01, MCL1) were also significantly modulated by dinaciclib (Supplementary Table S1), we hypothesised that the response of MB cells to dinaciclib was dependent on the modulation of the BCL2 family proteins, whose balance ultimately determines the fate of the mitochondrial apoptotic pathway^31^. To this aim, we first assessed the steady-state levels of BCL2, BCL-XL and MCL1 in HD-MB03 (Group 3) and ONS76 (SHH) cell lines by western blots. We found that both MCL1 and BAX were differentially expressed between the two cell lines (Fig. 6C). In accordance with the reduction of MCL-1 levels by dinaciclib in HD-MB03 and ONS76 cells (Fig. 5A), we investigated if MCL-1 down-regulation was in part accountable for dinaciclib-inhibitory effects in medulloblastoma. To this purpose, we genetically silenced *MCL-1* with two different siRNAs and observed a significant reduction in viability and a marked apoptotic response in both HD-MB03 and ONS76 cells (Fig. 6D,E and Supplementary Fig. S4B). Then, we used BCL-2 homology 3 (BH3)-mimetics as a pharmacological tool to alter the apoptotic balance of BCL2-family proteins in the MB cells. We subjected HD-MB03 and ONS76 cells to 72 h treatments with scalar concentrations of maritoclax (MCL-1 selective inhibitor), navitoclax (BCL-2, BCL-XL and BCL-W inhibitor) and WEHI-539 (BCL-XL selective inhibitor)^32–34^. We found that the MCL-1 selective inhibitor, maritoclax, was the most effective BH3-mimetic compound against MB cells. Interestingly, HD-MB03 and ONS76 cells were sensitive to the drug at nanomolar concentrations (Supplementary Fig. S4C).

Finally, we investigated if a combination therapy with BH3-mimics could strengthen the anti-proliferative effect of dinaciclib in MB cells. Since modulation of BCL-2 family members has been frequently associated to chemoresistance^35,36^, we explored two possible pharmacological strategies of combination with dinaciclib. On the one hand, we selected the specific MCL-1 inhibitor, maritoclax, to potentiate dinaciclib effect through the validated down-regulation of MCL-1; on the other hand, we chose navitoclax which inhibits BCL-2, BCL-XL and BCL-W but not MCL-1, to target possible compensatory anti-apoptotic mechanisms in MB. We treated HD-MB03 and ONS76 cells with scalar dinaciclib alone or in combination with either maritoclax or navitoclax for 72 h. MTT assay results showed that doses of maritoclax and navitoclax significantly enhanced dinaciclib growth inhibitory effects in both MB cell lines (Fig. 6F and Supplementary Fig. S4D). However, both BH3-mimetics were more efficient in HD-MB03 than ONS76 cells, since they synergised with lower doses of dinaciclib, corroborating the relevance of the higher MCL-1 levels in HD-MB03 cell line (Fig. 6F).

Overall, co-treatments of dinaciclib with MCL1 specific inhibitors have the potential to intensify treatment-response rate of MB cells and would reduce the concentration of each drug needed to elicit a satisfactory inhibitory effect. Furthermore, when used as a secondary line of treatment after current standard of care, these combinations could possibly tackle resistance insurgence in aggressive MB cells.

## Discussion

In this study we highlighted how the inhibition of multiple CDKs might be more beneficial for the treatment of MB than specific CDK4/6 inhibition, which is currently the only cell cycle targeted therapy pursued in clinical trials with palbociclib (NCT03709680). Starting from an unbiased in silico approach, we predicted that MB development and progression rely on druggable vulnerabilities that are not only limited to the G1/S checkpoint failure but also extended to the progression of the late stages of cell cycle and transcription regulation. Indeed, we singled out dinaciclib, a CDK1, CDK2, CDK5 and CDK9 inhibitor^37^, as the most effective CDKi across all MB cell lines, and specifically against the Group 3 HD-MB03 cells.

Our data show that dinaciclib, as a single agent, outperforms palbociclib in both 2D and 3D MB cell culture settings. We found that dinaciclib hampers cell proliferation and strongly induces cell death at nanomolar ranges in multiple medulloblastoma cells. Moreover, it impairs the stemness of these cells and, with our long-term studies, we showed that it ensures a prolonged cell death—up to 12 days from drug withdrawal—with the absence of outgrowing resistant colonies. On the contrary, palbociclib exerts only a cytostatic effect and long-term drug treatments allow the survival of MB cells, showing a limited inhibitory effect on MB stem cell population. These observations seem to confirm previous research showing resistance episodes in almost all treated solid tumours when palbociclib was withdrawn^14^ and are a call for concern when choosing palbociclib in MB context.

For the first time, we have characterised the molecular mechanisms of action of dinaciclib in MB cells. First, our in vitro studies showed a strong cytotoxic effect elicited by dinaciclib in HD-MB03 cells, with the main mechanism of cell death being the mitochondrial apoptotic pathway. We detected a caspase 3/7 activation in both adherent and 3D culture settings and, in parallel, cell fate profiles evidenced how MB cells die predominantly in interphase upon dinaciclib treatments. These results are in line with previous publications demonstrating that dinaciclib triggers the apoptotic cascade in multiple tumours, such as thyroid cancer, leukaemia and MYC-driven lymphoma^38–40^. We demonstrated that dinaciclib extensive apoptotic effects are mainly mediated by CDK9 inhibition and its consequential transcriptional inhibition of short-lived proteins, such as the anti-apoptotic MCL-1 and the transcriptional regulator MYC^26,40^. Indeed, using siRNA-mediated knockdown, we were able to induce apoptosis in multiple MB cells only by suppressing the levels of CDK9 but not with the repression of CDK1 or CDK2. With the use of BH3-mimic inhibitors targeting different BCL2 family members, we could confirm the unique role of MCL-1 as key mediator of cell death in those MB cells. In this context, MCL1 expression seems to act as a predictive biomarker for dinaciclib in MB cells; in fact, higher levels of MCL-1 protein in HD-MB03 cells were associated with higher drug responsiveness (tenfold difference) than ONS76 cells. This observation is in agreement with a previous study from Booher and co-authors that showed the predictive role of MCL-1 for dinaciclib sensitivity in other solid tumours^26^.

The role of MCL-1 to drive dinaciclib response in MB cells was further reinforced by the finding that BH3-mimetics boost its cytotoxic effects in MB cells, a strategy that has already been successful in previous research^41^. Since dinaciclib mechanism of action is mainly interfering with MCL-1 mediated apoptotic pathway, we selected maritoclax in an attempt to shut down MCL-1 activity completely, and navitoclax to limit compensatory BCL2 members activity, a mechanism that is commonly described as a form of resistance to MCL-1 inhibitors in cancer^42^. We demonstrated that combinatorial strategies with BH3-mimetics effectively synergise with dinaciclib and could be an additional option to improve therapeutic strategies for MB, although more detailed research is needed to determine their precise mechanism of action and envision administration protocols for patients.

Of note, CDK9 targeting by dinaciclib in MB also mediates the degradation of the oncogenic MYC, as has already been observed in different tumour settings^26,27^. This is particularly valuable for the treatment of Group 3 MB patients, whose tumours are shaped by MYC transcriptional programme towards the most aggressive MB phenotypes^43^. Furthermore, CDK9 has been reported as a druggable vulnerability in other MYC-dependent tumours^44,45^. Therefore, a stronger transcriptional impact on MYC in HD-MB03 cells might partially explain the higher sensitivity of Group 3 subgroup to the drug when compared with the other MB subgroups.

Our transcriptomic data offered further insights into the apoptotic molecular mechanisms elicited by dinaciclib in MB. On the one hand, it confirms the mitochondrial involvement for the significant upregulation of CASP9 mRNA after dinaciclib treatment, a known initiator caspase in the intrinsic apoptotic pathway^46^. On the other hand, it seems to exclude BAX involvement as a critical regulator for BCL-2 family inactivation in MB, since we observed downregulation of its mRNA and protein levels after dinaciclib treatment. A recent publication from *Xiaoou Xu *et al*.* provides a possible explanation for this, showing in melanoma cells how dinaciclib is able to induce apoptosis also in a BAX-independent manner via BAK^47^. Data showing that MCL-1 can bind alternatively to either BAX or BAK^48^ further suggest that BAK might have a crucial role in mitochondrial apoptosis induction by dinaciclib. However, further mechanistic studies are required to confirm this hypothesis in MB cells.

If CDK9 inhibition represents the main molecular switch that drives the apoptotic response of MB cells to dinaciclib, growth inhibitory effects may also be ascribed to CDK2 inhibition and to its negative control over the G1/S stage, as evidenced by the strong downregulation of the phosphorylation of pRB upon dinaciclib treatments. However, we were unable to observe any anti-proliferative activity in MB cells when we silenced CDK2 protein. Since, the decrease in pRB may also be associated to the direct inhibition of CDK5 that, despite its unconventional role^49^, has been found able to directly suppress the phosphorylation of pRB and affect the cell cycle^50^, we can speculate that the concurrent targeting of both CDK2 and CDK5 might be required for the effects on the RB pathway. Furthermore, the inhibition of CDK5 by dinaciclib has already been shown and concurrently reported as critical player for its anti-tumour immune response in vivo^51^. This hints at the potential benefits of using this broad-CDKi for MB treatment for its meddling role in multiple processes such as cell cycle, apoptosis and immunosurveillance. Collectively, this study provides promising pre-clinical evidence on the better efficacy of dinaciclib over palbociclib for MB treatment. A major cause for concern when using palbociclib in the clinic is the onset of resistance, mainly due to its narrow CDK targeting and re-wiring of the cell cycle checkpoints^52^. Dinaciclib holds great potential in the group of CDKis because, with its broad inhibitory spectrum, it can dampen the onset of parallel resistance mechanisms. Specifically, we showed that targeting of the transcriptional CDK9 should be a priority in MB treatment, due to its ability to downregulate downstream oncogenic pathways (MYC) and trigger the apoptotic response (MCL-1). Dinaciclib has now reached phase III clinical trials for many malignancies, showing encouraging anticancer activity; however, monotherapy with dinaciclib is still under consideration due to unwanted toxicities. To address this point, we envision that combination therapies with dinaciclib may provide a safer and more effective solution for MB patients, preventing common forms of drug-resistance and attenuating treatment side effects.

## Materials and methods

### Cell culture

MB cell lines DAOY (SHH MB) and CHLA-01-MED (Group 4 MB) were purchased from ATCC and HD-MB03 (Group 3 MB), ONS76 and UW228 cells (SHH MB) were a kind gift from Dr. Caroline Springer. All cell lines were maintained in RPMI-1640 (Biosera) supplemented with 10% (v/v) Fetal Bovine Serum and 1% (v/v) Penicillin–Streptomycin (Gibco), except for CHLA-01-MED which were grown in DMEM:F12 medium with 20 ng/mL human recombinant EGF, 20 ng/mL human recombinant basic FGF, and B-27 Supplement (Invitrogen) to a final concentration of 2% (v/v).

All cell lines were incubated at 37 °C and 5% CO_2_ and passaged every 3 days upon confluency.

### 3D spheroid culture

HD-MB03 3D spheroids were generated with a forced floating strategy, seeding 500 cells/well in the Nunclon Sphera 96-well microplate with super low cell attachment surface (ThermoFisher). The plate was then centrifuged at 200*g* for 2 min, to gather all cells at the bottom of each well. Cells were incubated at 37 °C and 5% CO_2_ for 5 days to allow spheroids formation prior to treatment. Spheroid formation was assessed through phase-contrast microscopy with BioTek Cytation 3 Image reader (BioTek).

Spheroid volume was measured using the following formula $\frac{Lenght\times {Width}^{2}}{2}$^53^.

### Compounds used

All CDKis were purchased from MedChemExpress and diluted in DMSO. CDKis were used with a 0–10 μM concentration range for in vitro treatments.

The BH3-mimetic compounds were obtained from APExBIO. Specifically, we used the BCL-XL, BCL-2 and BCL-w inhibitor navitoclax, the selective MCL-1 inhibitor maritoclax, and the selective BCL-XL inhibitor WEHI-539. All BH3-mimetics were tested at 0–100 μM range of concentrations.

All working drug solutions were stored at − 20 °C.

### Transfection

MB cells were cultured to 50–60% confluence at 200,000 cells/well in 6-well plates or 1500–2000 cells/well in 96-well plates in antibiotics-free RPMI medium for 24 h. Cells were transfected with 50–100 nM final concentration of specific siRNA for 72 h. The day after transfection, culture medium was changed to avoid lipofectamine toxicity effects on cells.

All transfections were performed using Lipofectamine 2000 or Lipofectamine RNAiMax according to manufacturer’s instructions (Invitrogen).

A pool of 4 different siRNAs (SMARTpool) for non-targeting siRNA control, CDK1, CDK2, CDK9 were purchased from Dharmacon and single siRNAs for MCL-1 and siRNA control were obtained from Qiagen.

### Short-term cell proliferation assays

MB cells were seeded in 96-well plates at 1000 cells/well DAOY, 2000 cells/well HD-MB03, 2500 cells/well UW228, 1200 cells/well ONS76 and 5000 cells/well CHLA-01-MED. Next day, cells were treated with scalar concentration of selected drugs for 72 h.

Short-term viability of adherent MB cells was determined after different treatment conditions in quadruplicates and examined by Thiazolyl Blue tetrazolium bromide (MTT assay) (Alfa Aesar), whereas suspension MB cells viability was measured by Alamar blue (Alfa Aesar) resazurin assay. Formazan solution production from MTT was measured at 540 nm, whereas resazurin reagent absorbance was detected at 600 nm by absorbance in the FLUOstar Omega microplate reader (BMG Labtech). Absorbance was normalised to the untreated control, data were fitted using a non-linear regression curve and IC_50_ values for each compound were calculated (Prism 5 software, GraphPad).

### Long-term cell proliferation assays

Long-term viability assays were performed in two ways: on the one hand, by seeding 200 cells in 96-well plates and, at 24 h from seeding, treating cells in quadruplicates every 72 h for 9 days with selected drugs.

On the other, wash-out experiments were performed by seeding 200,000 cells/well in 6-well plates. Cells were cultured with increasing concentrations of selected drugs for 24 h prior to wash-out. Then, cells were counted again and seeded for long-term analyses at 200 cells/well in 6-well plates. Cells were monitored over 2 additional weeks, with media replacement every 2 days.

At the endpoint of each long-term assay, survivor strains were stained with crystal violet solution (0.5% w/v, Sigma-Aldrich) for 20 min on a bench rocker at room temperature, rinsed with water and air-dried overnight. Crystal violet staining was dissolved by adding 200 μl of methanol to each well. The optical density of crystal violet solution was measured using FLUOstar Omega reader (BMG Labtech) at 570 nm. Absorbance values were analysed with GraphPad Prism 5 software.

### Tumour sphere assay

HD-MB03 cells were first treated with scalar concentrations of dinaciclib and palbociclib for 72 h. A single cell suspension was then prepared using trypsinisation and manual disaggregation with a 25-gauge needle. 5000 cells/well were plated in low adhesion 6-well plates and cultured in Neural Precursor Media: DMEM/F12 (ThermoFisher) containing 10 ml of B27 (50 ×), 20 ng/ml EGF, and 20 ng/ml bFGF (ThermoFisher). Low adhesion plates were prepared by coating each well with 1.5 ml of 1.2% Poly 2-hydroxyethyl methacrylate (PolyHema, Sigma). After 5 days in culture, spheres were visualised and counted using an eye piece graticule in an inverted microscope (Zeiss Primovert). Spheres were counted only for diameters of at least 50 µm. Tumour sphere assay was always performed in three technical replicates and repeated three times independently.

### Apoptotic assay

Caspase 3/7 activity after 72 h siRNAs transfections was measured using the Caspase-Glo 3/7 Assay system (Promega) under manufacturer’s instructions. Luminescence was measured using the Varioskan microplate reader (Thermo Scientific). All results were normalized to non-targeting control siRNA values.

Caspase 3/7 activity assessment after Dinaciclib and Palbociclib treatments was performed using CellEvent Caspase-3/7 Green Detection Reagent (ThermoFisher) in 2D and 3D MB cell cultures. Briefly, for 2D treatments, HD-MB03 cells were plated at 3500 cells/well in 96-well plates, and for 3D experiments, spheroids were generated as previously described. In both culture settings, cells were treated with 100 nM of the CDKis tested for 24 h. Then, Caspase-3/7 reagent was added to culture media at 3 μM final concentration and incubated for 30 min before imaging. Fluorescence from caspase-active cells was detected with a BioTek Cytation 3 Image reader (BioTek).

### Cell cycle analysis

Cells were plated in 6-well plates at 250,000 cells/well and 24 h later were treated with selected CDKis or DMSO control. At 48 h from drug treatment, cells were trypsinized, washed with PBS, and fixed with ice-cold 70% ethanol for 30 min while being vortexed. Cells were washed twice with ice-cold PBS before flow-cytometric analysis. First, 50 μl of 100 μg/ml RNAse A was added to each sample cell pellet and incubated for 15 min at 37 °C to avoid RNA staining contamination. Then, cells were labelled with 200 μl of propidium iodide (PI) solution (50 μg/ml) (Sigma-Aldrich) for 15 min at room temperature.

20,000 events per treatment were recorded using BD FACSVerse flow cytometer (BD Biosciences), and data analysed using the BD FACSuite software (BD Biosciences). Cell cycle plots were generated using GraphPad Prism 5 software as percentage of gated cells in each phase of the cell cycle.

### Time-lapse microscopy

Cells were seeded at 2500 cells/well in 96-well plates and treated the next day with scalar doses of dinaciclib. Each treatment condition was performed in technical quadruplicates. The plate was then maintained at 37 °C and 5% CO_2_ in the Cytation 3 system (Biotek). Phase-contrast images were recorded every 15 min for 48 h at 100 × magnification. Data acquisition was carried out with the Gen5 software. Changes in cell morphology were assessed by visually analysing the fate of at least 25 cells per condition, across the 4 replicate wells. At the beginning of each video a single cell in interphase was selected and its cell cycle progression analysed by manually tracking the cell, frame by frame. Following mitosis, one of the daughter cells was randomly chosen and tracked further.

Finally, timing results and statistical analysis were performed using GraphPad Prism 5.

### Western blot analysis

Proteins were extracted using a Triple Lysis Buffer composed of 50 mM Tris–HCl (pH 7.5), 150 mM NaCl, 0.1% SDS, 1% NP-40 and 0.5% sodium deoxycholate, with the addition of Protease Inhibitor Cocktail set III and Phosphatase Inhibitor Cocktail set II (EMD Millipore). Protein content was determined with Bradford assay (Bio-Rad).

Equal amounts of protein lysates (30–50 μg) were resolved by 4–20% SDS-PAGE, transferred to 0.45 μm nitrocellulose membranes (Bio-Rad) and blocked in 5% (w/v) skimmed milk in PBS containing 0.1% (v/v) Tween-20 (Bio-Rad) for 1 h at room temperature. After blocking, membranes were incubated overnight at 4 °C with the indicated antibodies. Blots were then incubated at room temperature for 1 h with secondary HRP-conjugated antibodies (Amersham) and bands were revealed in SuperSignal West Pico/Dura/Fempto Chemiluminescent Substrate (Thermo Fisher). Chemiluminescence was recorded in a ChemiDoc imaging system (Bio-Rad). A list of all antibodies used can be found in the Supplementary Table S2. GAPDH, Actin,Tubulin or Vinculin were used as loading controls.

### RNA-Seq analysis

HD-MB03 cells were treated with DMSO or 1 nM dinaciclib for 24 h. All treatment conditions were submitted and processed in triplicates at the Sequencing facility of the Cancer Research, UK. Sequencing libraries were prepared by using the QuantSeq kit (Lexogen) and run on an Illumina NextSeq sequencer.

A sequential alignment procedure was performed to map raw reads against the GRCh38 Human Genome reference (release 83). First, all reads were aligned by ‘STAR’^54^; then, using ‘Bowtie2’^55^, we locally mapped those reads discarded by STAR. Gene expression quantification was computed by ‘featureCounts’^56^. The ‘DaMiRseq’ Bioconductor/R package was used to filter out genes (less than 10 counts in more than 50% of samples), perform the normalization (variance stabilizing transformation), and search for confounding factors^57^. Differential analysis was performed by the ‘limma’ R/Bioconductor package^58^. A gene was deemed significant whether the *P* value was < 0.05 and logFC threshold of 0.5. A Benjamini–Hochberg procedure was performed to control the false discovery rate (FDR).

### Gene Ontology enrichment analysis

Gene ontology enrichment analysis (GO) (http://geneontology.org) for differentially expressed genes (*P* value < 0.05 and logFC threshold of 0.2) was perfomed by using the GO tool powered by PANTHER. Relevant pathways were selected based on FDR < 0.05 and Fold Enrichment > 1.5. RNA-seq datasets. Publicly available dataset of Cavalli et al. (2016) from the R2: Genomics Analysis and Visualization Platform (https://hgserver1.amc.nl/cgi-bin/r2/main.cgi) was used to retrieve all cell cycle related genes that were associated to the overall survival of medulloblastoma patients. The cut-off for the final list of genes was *P *value < 0.05 and results were corrected by FDR.

### Quantitative PCR (qPCR)

Total RNA was isolated using TRIzol Reagent (Ambion), according to the manufacturer’s instructions. 500 ng of RNA was reverse transcribed with Superscript III First-Strand Synthesis kit (Thermo Fisher). Then, 1 μl of diluted cDNA (50 ng/μl) from each reaction was amplified with the FS Universal SYBR Green Master Rox master mix (Roche) as per manufacturer’s protocol. All reactions were run on a Lightcycler 96 instrument (Roche).

Relative expression was calculated using the comparative Ct method (2-ΔΔCt)^59^. Specifically, β-actin served as housekeeping gene to normalise relative expression of genes of interest.

All the primers used were custom-designed and can be found in Supplementary Table S2.

### Statistical analysis

Results are shown as mean ± SD from 3 independent replicates for all experiments unless stated otherwise in the figure legend. The analyses were performed using GraphPad Prism software version 7 (GraphPad Software, Inc, La Jolla, CA, USA). A value of *P* < 0.05 was considered statistically significant. Statistical significance was determined using a Student’s t-test where *P < 0.05; **P < 0.01; ***P < 0.001; N.S. = Not Significant *P* > 0.05.

Synergy degree of drug combinations was determined by Chou and Talalay CI method with the CompuSyn software^60^ where CI values < 1 indicate synergistic interactions, CI = 1 additive interactions and CI > 1 antagonistic interaction.

## Supplementary Information

Supplementary Table S1.

Supplementary Table S2.

Supplementary Figures.
