# Author Correction: Pre-therapeutic efficacy of the CDK inhibitor dinaciclib in medulloblastoma cells

**DOI:** 10.1038/s41598-024-83271-0

**Published:** 2025-02-05

**Authors:** Marta Buzzetti, Sonia Morlando, Dimitrios Solomos, Ammara Mehmood, Alexander W. I. Cox, Mattia Chiesa, Yuri D’Alessandra, Michela Garofalo, Caroline H. Topham, Gianpiero Di Leva

**Affiliations:** 1 School of Science, Engineering and Environment, Biomedical Research Centre, Salford, Greater Manchester UK; 2 Transcriptional Networks in Lung Cancer Group, Cancer Research UK Manchester Institute, University of Manchester, Manchester, UK; 3 Bioinformatics and Artificial Intelligence Facility, Centro Cardiologico Monzino IRCCS, Milan, Italy; 4 Immunology and Functional Genomics Unit, Centro Cardiologico Monzino IRCCS, Milan, Italy; 5 Cancer Research UK Lung Cancer Centre of Excellence, at Manchester and University College London, London, UK; 6 School of Pharmacy and Bioengineering, Guy Hilton Research Centre, Keele University, Stoke-on-Trent, UK

Correction to: *Scientific Reports* 10.1038/s41598-021-84082-3, published online 08 March 2021

The original version of this Article contained an error in Figure 2, panel C. During Figure preparation, the NT control well in the Palbociclib condition was mistakenly duplicated from the Dinaciclib condition. The original Fig. 2 and accompanying legend appear below.

**Fig. 2:**
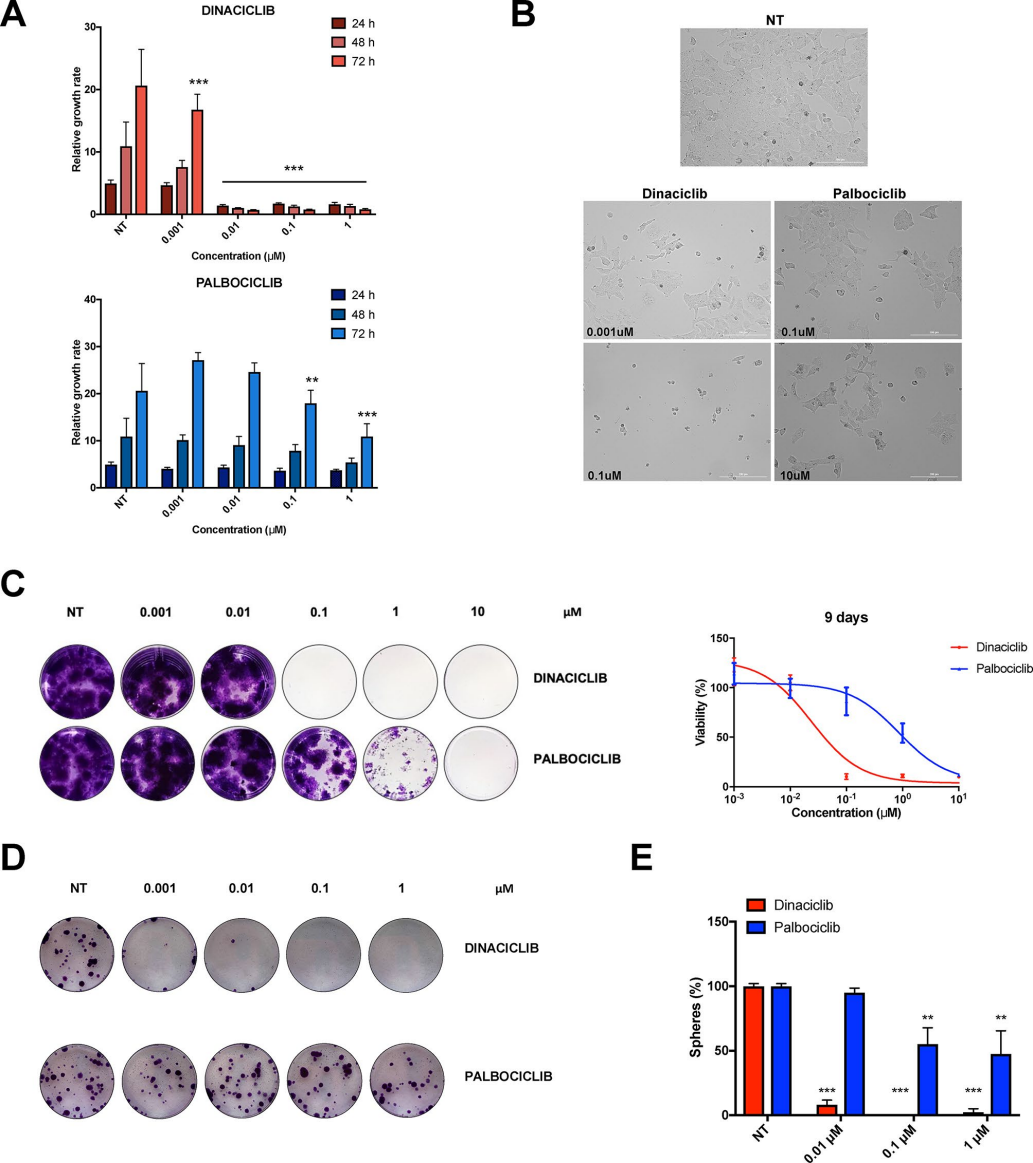
Dinaciclib shows stronger inhibitory responses than palbociclib in medulloblastoma. (**A**) Growth rates over 72 h of HD-MB03 cells treated with dinaciclib (top) or palbociclib (bottom) at the indicated concentrations. (**B**) Representative phase-contrast images of HD-MB03 cells treated with IC50 and 100xIC50 doses of dinaciclib and palbociclib for 72 h. Images were captured at 100 × total magnification. (**C**) Representative crystal violet staining images of long-term proliferation assay. HD-MB03 cells were treated with the indicated drugs for 9 days and then fixed and stained. On the right, crystal violet quantifications relative to the presented images. (**D**) Representative crystal violet staining images showing resistant HD-MB03 colonies arising after dinaciclib and palbociclib wash-out over 12 days recovery. Pre-treatments with each drug were carried out over 24 h before wash-out. (**E**) Percentages of cell viability inhibition of HD-MB03 medullospheres after 72 h treatment with different doses of dinaciclib or palbociclib, calculated as relative to untreated control. Statistical comparisons were performed using an unpaired, two-tailed Student t-test where **P < 0.01; ***P < 0.001. Plotted graphs show mean ± SD (n = 3).

The original Article has been corrected.

