# Supplementary material for: Pre-therapeutic efficacy of the CDK inhibitor dinaciclib in medulloblastoma cells
Source: Sci Rep. 2021 Mar 8;11:5374. doi: 10.1038/s41598-021-84082-3 (PMC7940474; doi:10.1038/s41598-021-84082-3)
Supplement: Supplementary file 3 — Supplementary Figures. [file 41598_2021_84082_MOESM3_ESM.pdf]

Pre-therapeutic efficacy of the CDK inhibitor dinaciclib in medulloblastoma cells

Marta Buzzetti[1,2,7], Sonia Morlando[1,7], Dimitrios Solomos[1], Ammara Mehmood[1], Alexander W I Cox[5], Mattia Chiesa[3], Yuri D'Alessandra[4], Michela Garofalo [2,5], Caroline H Topham[1] and Gianpiero Di Leva[6,*]

Affiliations

[1] Biomedical Research Centre, School of Science, Engineering and Environment, Salford, Greater Manchester, UK.

[2] Transcriptional Networks in Lung Cancer Group, Cancer Research UK Manchester Institute, University of Manchester, Manchester, UK.

[3] Bioinformatics and Artificial Intelligence facility, Centro Cardiologico Monzino IRCCS, Milan, Italy.

[4] Immunology and Functional Genomics Unit, Centro Cardiologico Monzino IRCCS, Milan, Italy.

[5] Cancer Research UK Lung Cancer Centre of Excellence, at Manchester and University College London, London, UK.

[6] Keele University, School of Pharmacy and Bioengineering, Guy Hilton Research Centre, Stoke-on-Trent, UK.

[7] These authors contributed equally to the manuscript.

[*] Correspondence should be addressed to: g.dileva@keele.ac.uk

CDK Inhibitor	CDK1	CDK2	CDK4	CDK5	CDK6	CDK7	CDK9
AT7519	++	++	++	+++	++	+	+++
Dinaciclib	++++	++++		++++			++++
Flavopiridol	+++	+++	+++				
LEE011			+++		+++		
Milciclib	+	++	++	+		++	
Palbociclib			++++		+++		
PHA-767491	+	++		+			+++

Supplementary Fig. S1

Supplementary Fig. S1:

Different selectivity of CDKis used in the study. + symbol shows which CDKs are targeted by each compound as they are indicated by the manufacturer.

A

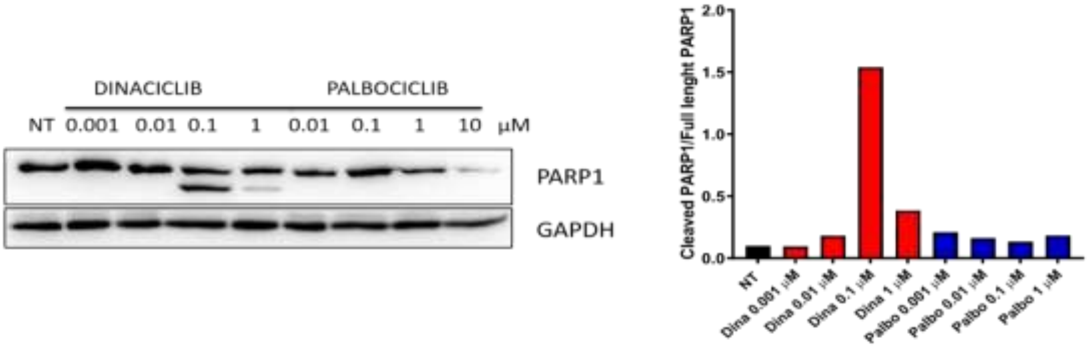

Supplementary Fig. S2

Supplementary Fig. S2:

A) HD-MB03 cells were treated with indicated doses of dinaciclib and palbociclib for 24 hrs. Cell extracts were analysed by Western blotting. On the right, densitometric analysis of presented blots (n=3).

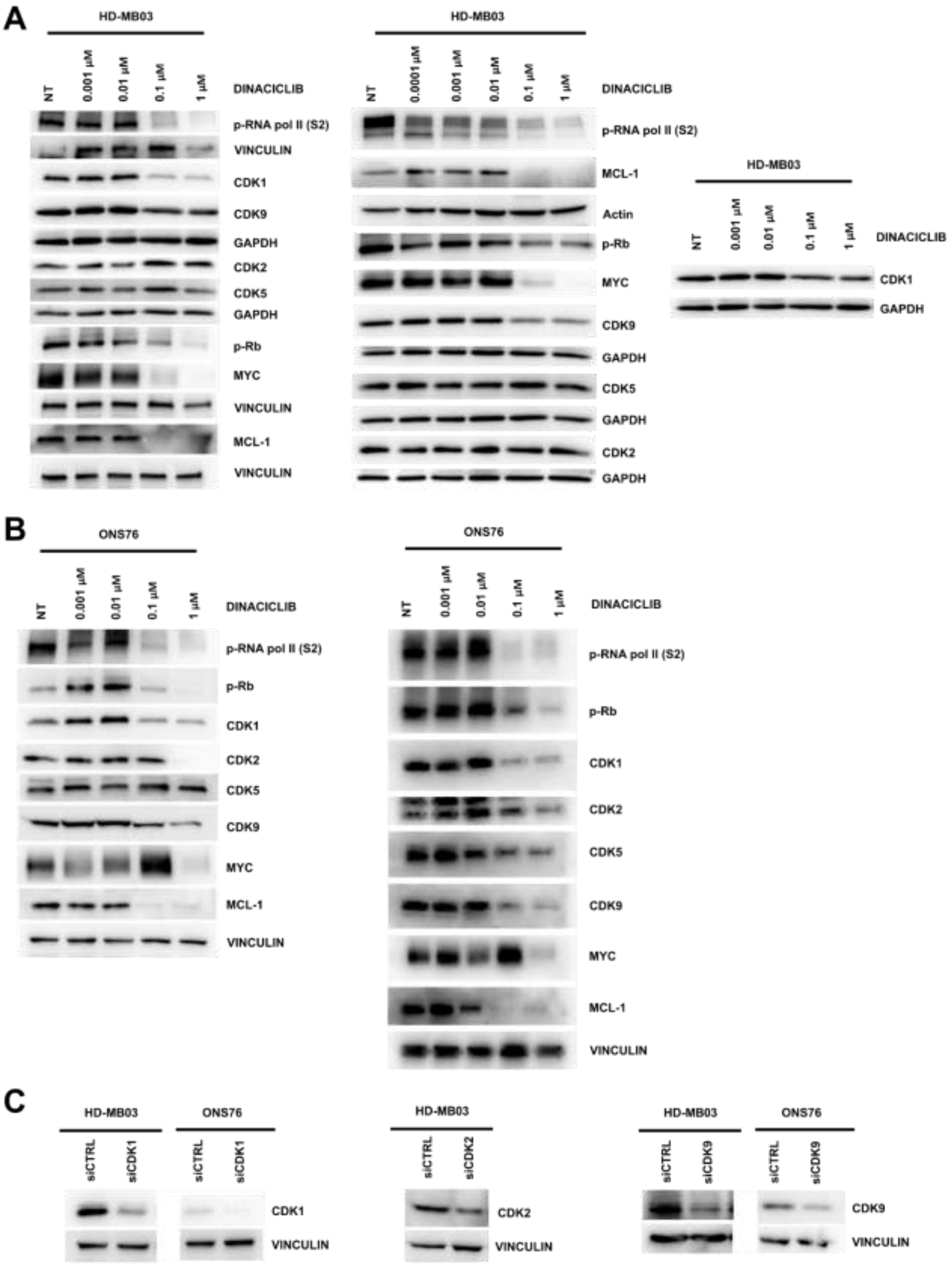

Supplementary Fig. S3

Supplementary Fig. S3:

A) Western blot replicates for dinaciclib treatments on HD-MB03 cells used for densitometric analyses shown in Figure 5B (n=3).

B) Western blot replicates for dinaciclib treatments on ONS76 cells used for densitometric analyses shown in Figure 5D (n=3).

C) Representative western blot analysis of HD-MB03 cells and ONS76 cells treated with siCTRL or siRNA for CDK1, CDK2 or CDK9 for 72 h.

A

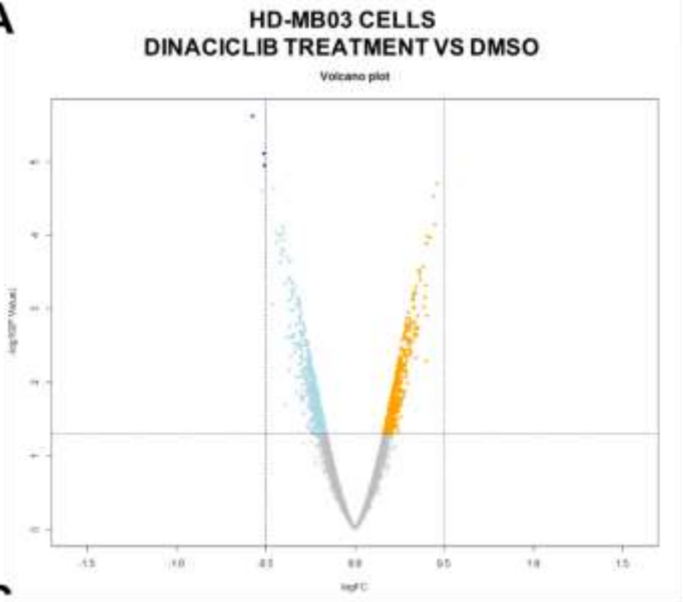

B

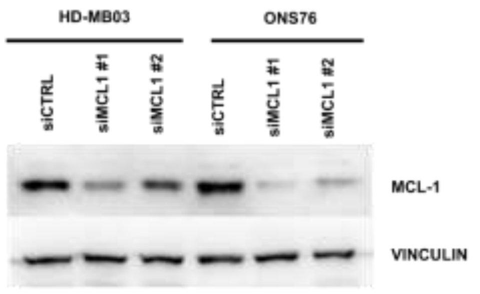

C

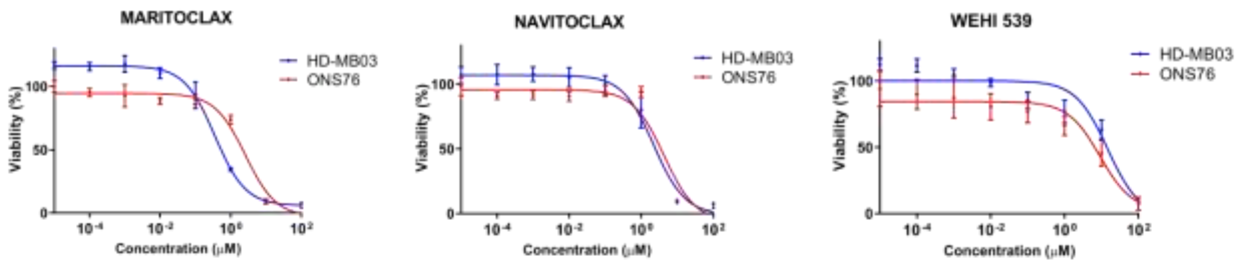

	HD-MB03	ONS76
IC50	0.3562	2.604

	HD-MB03	ONS76
IC50	2.213	4.319

	HD-MB03	ONS76
IC50	14.70	9.112

D

HD-MB03

Maritoclax (μM)

Dinaciclib (μM)	0.01	0.1	1	10
6.10E-5	2.53E16	>10	0.02 ***	0.001 ***
2.44E-4	2.53E16	>10	0.17 ***	0.003 ***
9.77E-4	0.09 **	>10	0.14 ***	0.006 ***
0.004	0.09	0.29	0.42 ***	0.03 ***
0.016	0.10	0.10	0.09	0.09
0.062	0.38	0.39 *	0.37	0.34
0.25	1.46	1.45	1.47	1.48
1	6.1	5.83	5.7	5.84

Navitoclax (μM)

Dinaciclib (μM)	0.01	0.1	1	10
6.10E-5	>10	>10	0.003 **	0.01 ***
2.44E-4	1.13	>10	0.01 *	0.01 ***
9.77E-4	6.6	>10	0.02 *	0.01 ***
0.004	0.07	0.09	0.06	0.03 ***
0.016	0.11	0.11	0.10	0.08 **
0.062	0.31	0.31	0.30	0.32
0.25	1.30	1.27	1.20	1.24
1	5	5	5	5

ONS76

Maritoclax (μM)

Dinaciclib (μM)	0.01	0.1	1	10
6.10E-5	>10	>10	>10	2.43E-4 ***
2.44E-4	>10	0.15	>10	8.80E-4 ***
9.77E-4	>10	0.85	>10	0.004 ***
0.004	0.8 **	0.53 **	>10	0.01 ***
0.016	1.47	0.66	0.58	0.05 ***
0.062	0.18	0.18	0.19 *	0.12 ***
0.25	0.74	0.67 *	0.69 *	0.52 *
1	2.9	2.79	2.80	2.24

Navitoclax (μM)

Dinaciclib (μM)	0.01	0.1	1	10
6.10E-5	>10	>10	>10	0.002 ***
2.44E-4	>10	>10	>10	0.003 ***
9.77E-4	>10	>10	>10	0.01 ***
0.004	>10	6.32	0.06	0.01 ***
0.016	1.47	1.14	0.7	0.01 ***
0.062	0.04	0.03 **	0.04 ***	0.03 ***
0.25	0.22	0.09 ***	0.15 ***	0.14 ***
1	0.92	0.51	0.57 ***	0.51 ***

Supplementary Fig. S4

Supplementary Fig. S4:

A) Volcano plot representation of the RNAseq data performed in HD-MB03 untreated (DMSO) vs HD-MB03 cells treated with 1nM dinaciclib for 24 hrs. Blue dots indicate all downregulated genes and orange dots indicate upregulated genes upon treatment (logFC±0.5, p-value < 0.05). Volcano plot has been generated using R software (https://www.R-project.org/). **B)** Representative western blot analysis of HD-MB03 cells and ONS76 cells treated with siCTRL or two different siRNAs for MCL-1 for 72 h. **C)** Proliferation assays of HD-MB03 and ONS76 cells treated with maritoclax, navitoclax and WEHI-539 respectively. Below, IC50 values (μM) are presented. **D)** Combination index (CI) values for indicated drug combinations in HD-MB03 and ONS76 cells were calculated using CompuSyn software. CI values lower than 1 indicate synergistic combinations (green); CI values greater or equal than 1 indicate antagonistic combinations or additive combinations, respectively. Statistical comparisons were performed between the inhibitory effect of the combination treatment vs dinaciclib only treatment effect using a two-tailed Student's t-test where *P < 0.05, **P < 0.01, ***P < 0.001.

A

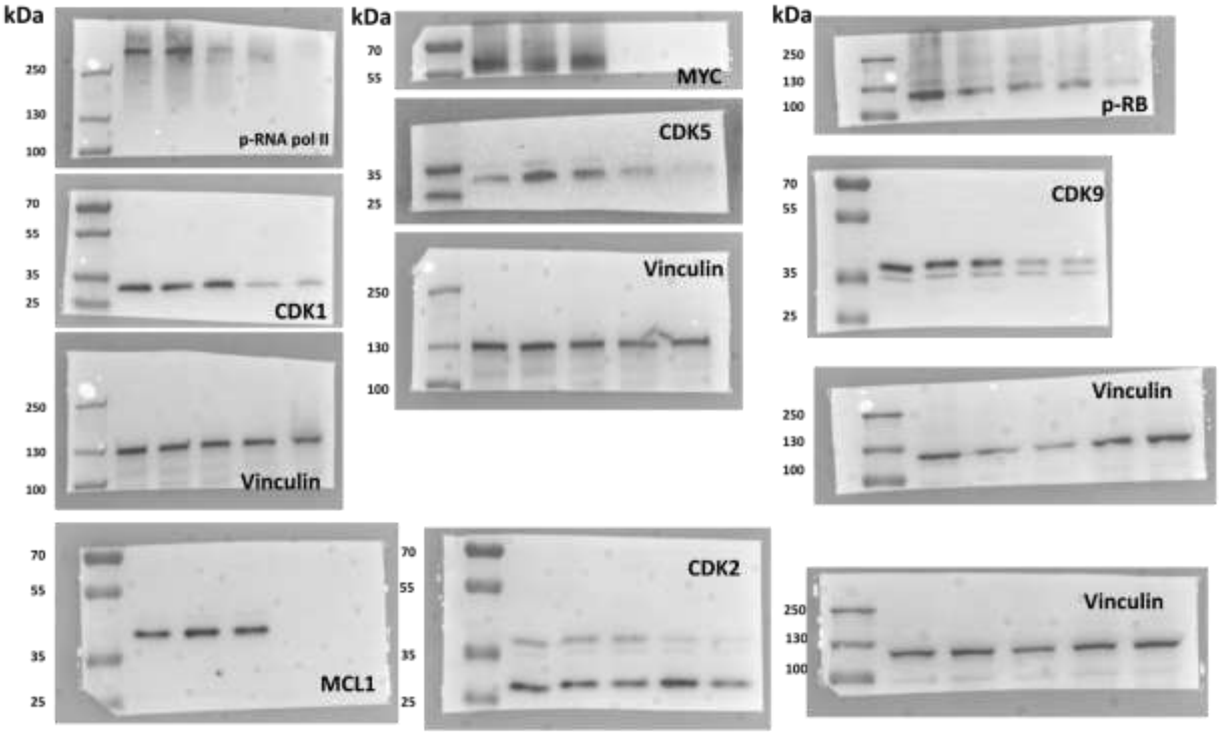

B

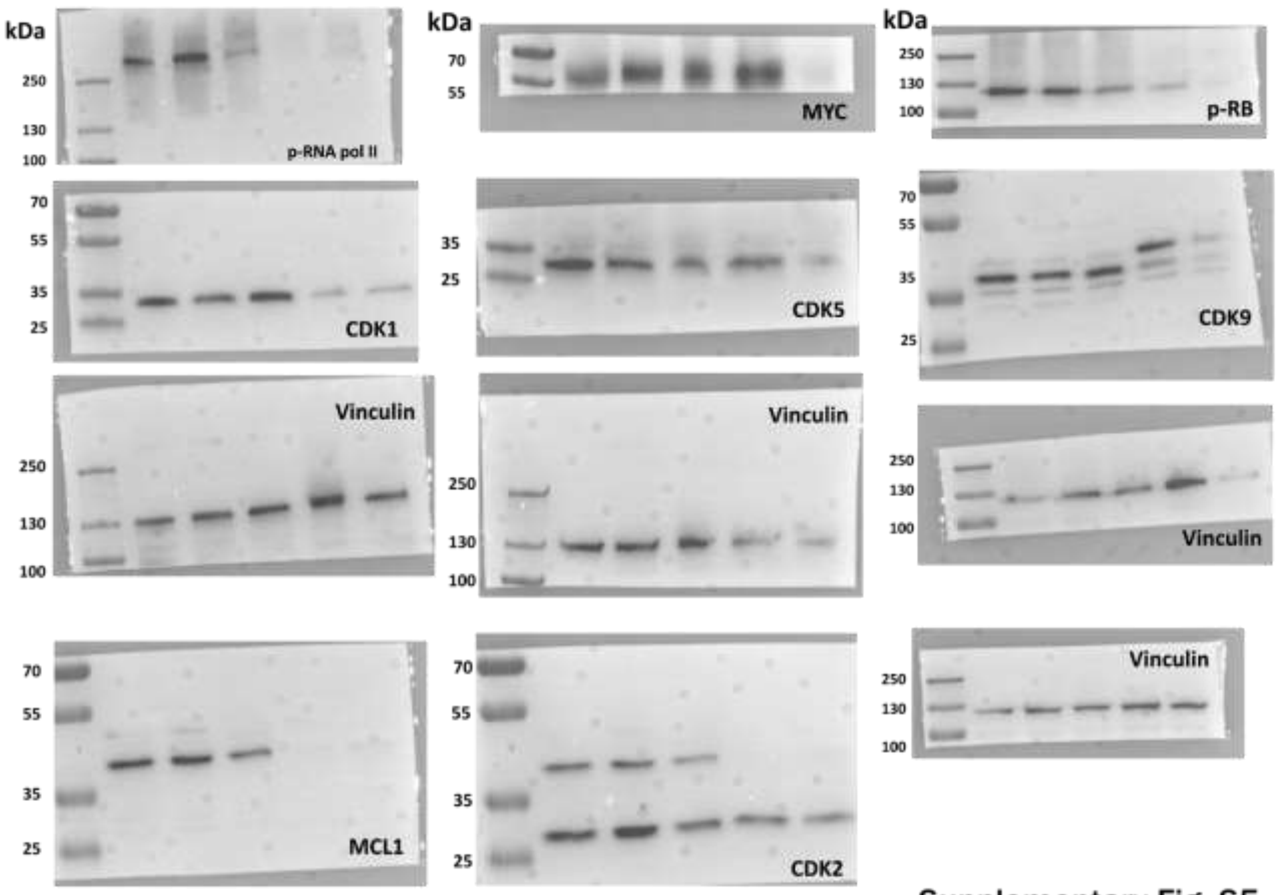

Supplementary Fig. S5

Supplementary Fig. S5:

A) Raw membranes of western blots shown in Figure 5A. **B)** Raw membranes of western blots shown in Figure 5C.

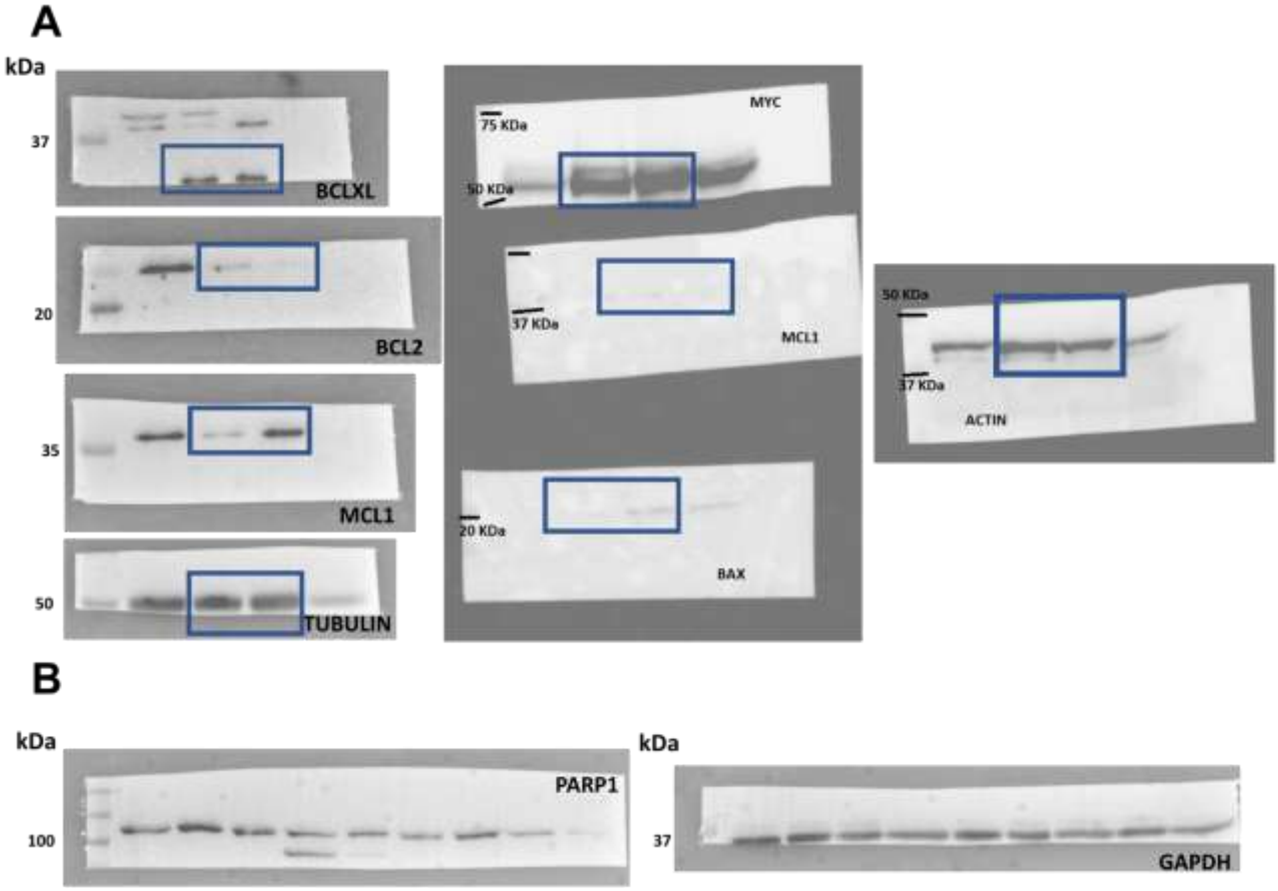

Supplementary Fig. S6

Supplementary Fig. S6:

A) Raw membranes of western blots shown in Figure 6C. **B)** Raw membranes of western blots shown in Supplementary Figure S2A.

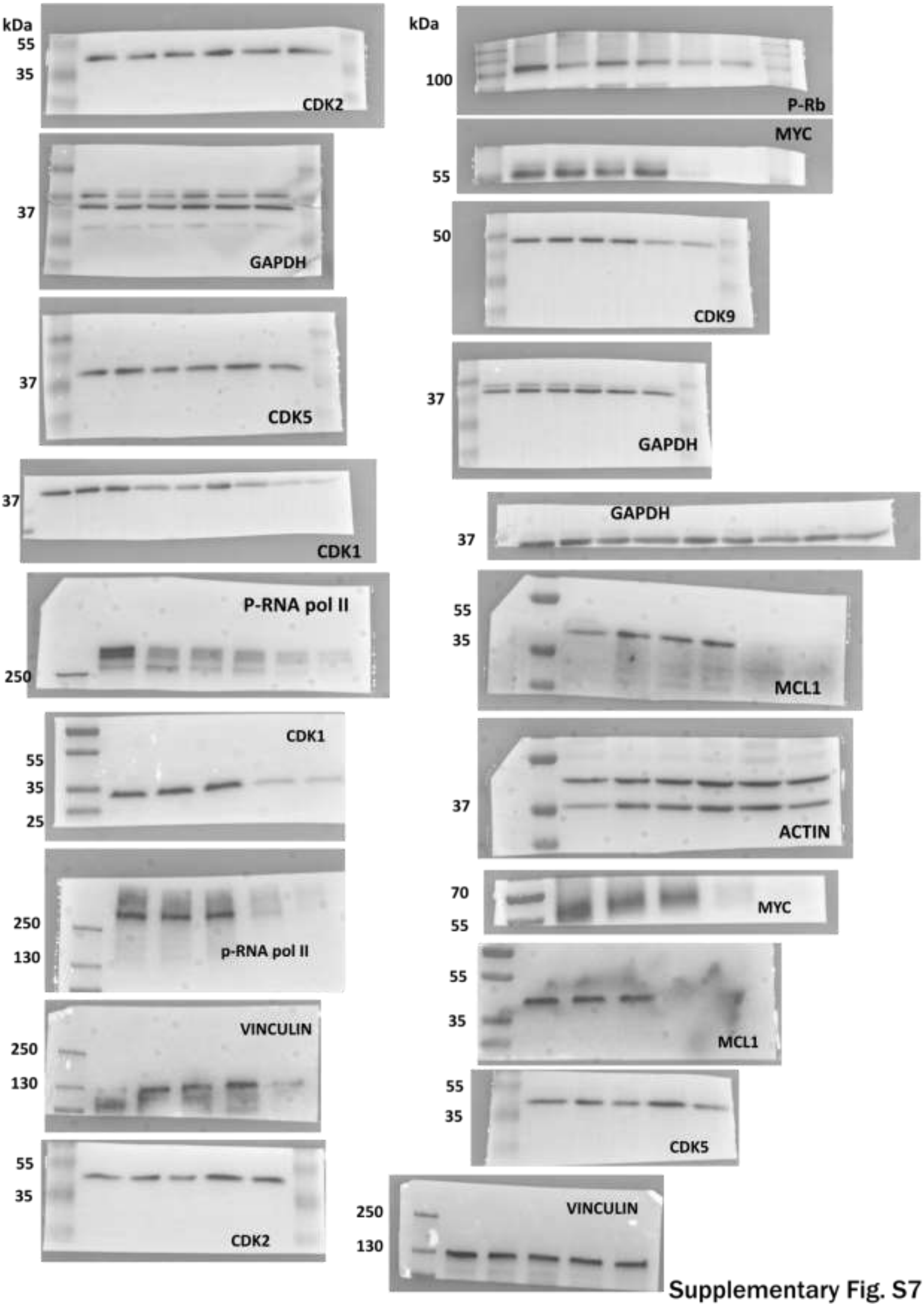

Supplementary Fig. S7

Supplementary Fig. S7:

Raw membranes of western blots shown in Supplementary Figure S3A.

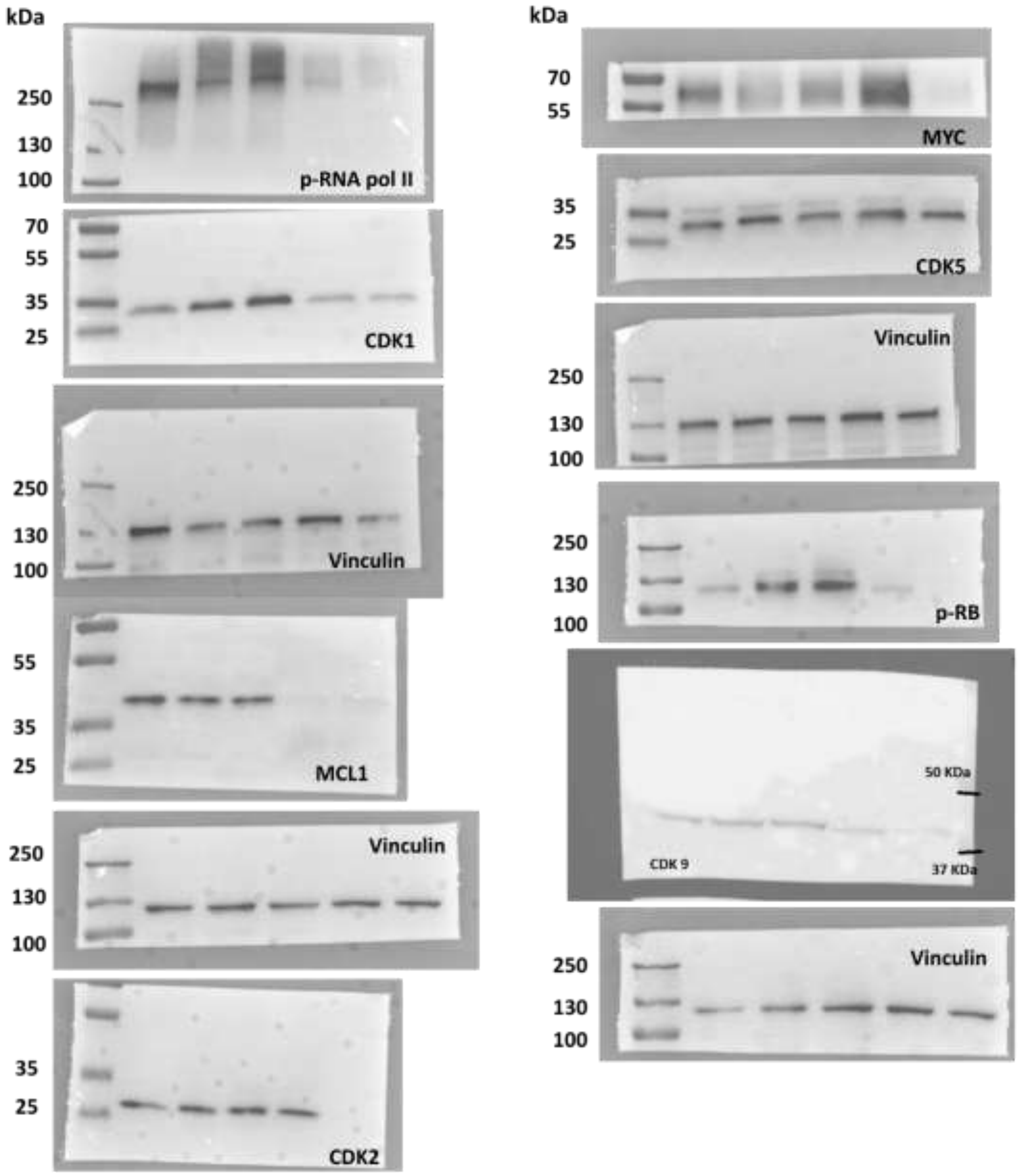

Supplementary Fig. S8

Supplementary Fig. S8:

Raw membranes of western blots shown in Supplementary Figure S3B.

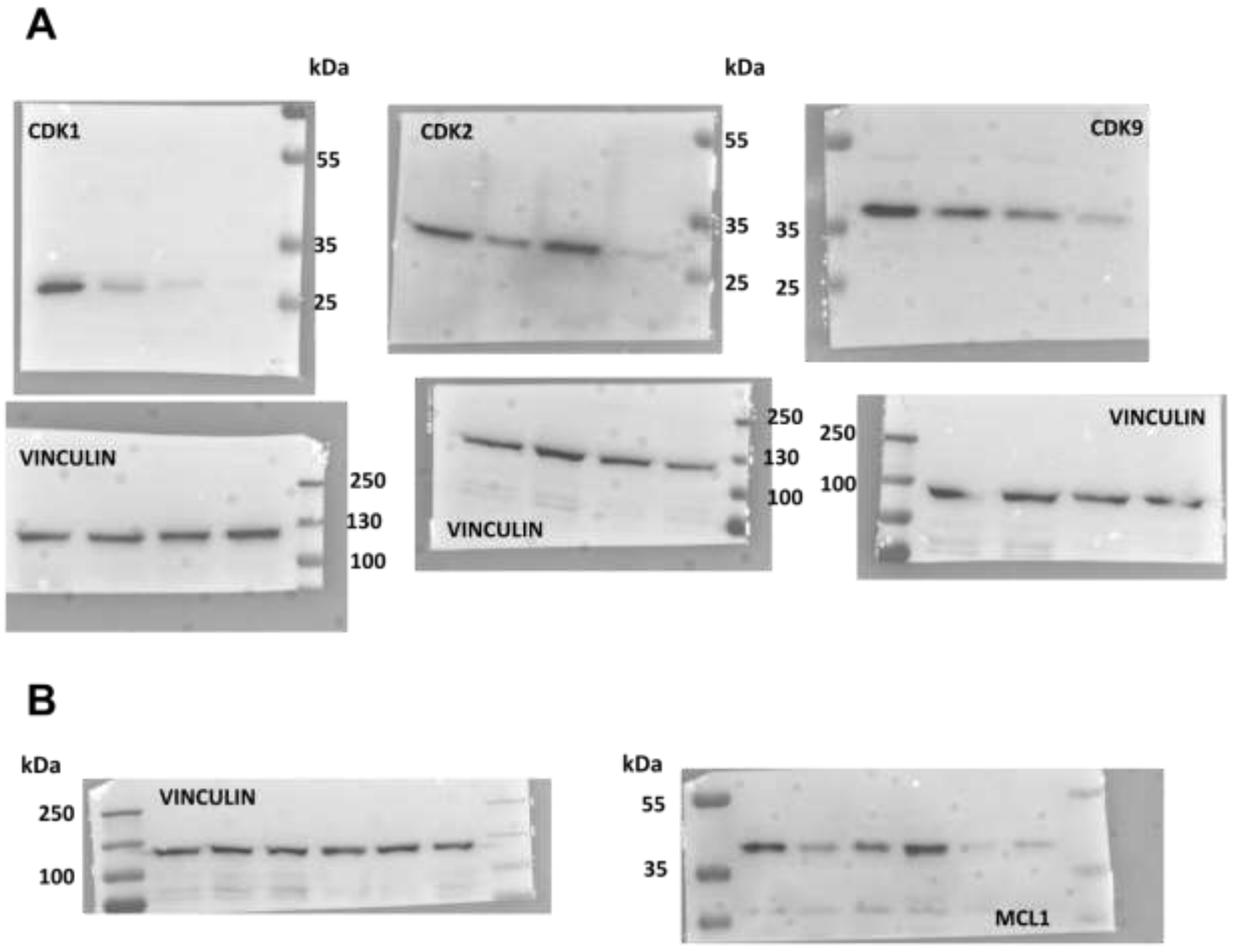

Supplementary Fig. S9

Supplementary Fig. S9:

A) Raw membranes of western blots shown in Supplementary Figure S3C. **B)** Raw membranes of western blots shown in Supplementary Figure S4B.
